# Time-Varying Pattern of Postoperative Recurrence Risk of Early-Stage (T1a-T2bN0M0) Non-Small Cell Lung Cancer (NSCLC): Results of a Single-Center Study of 994 Chinese Patients

**DOI:** 10.1371/journal.pone.0106668

**Published:** 2014-09-09

**Authors:** Jian-fei Zhu, Xing-yu Feng, Xue-wen Zhang, Ying-sheng Wen, Peng Lin, Tie-hua Rong, Ling Cai, Lan-jun Zhang

**Affiliations:** 1 Department of Thoracic Surgery, Sun Yat-sen University Cancer Center, Guangzhou, Guangdong, China; 2 Shaanxi Provincial People’s Hospital, Xi’an, Shaanxi, China; 3 Department of Gastro-pancreatic Surgery, Sun Yat-sen University Cancer Center, Guangzhou, Guangdong, China; 4 Department of Pathology, Sun Yat-sen University Cancer Center, Guangzhou, Guangdong, China; 5 Department of Radiation-Oncology, Sun Yat-sen University Cancer Center, Guangzhou, Guangdong, China; Memorial Sloan-Kettering Cancer Center, United States of America

## Abstract

**Background:**

The aim of this study was to analyze the time-varying pattern of recurrence risk of early-stage (T1a-T2bN0M0) non-small cell lung cancer (NSCLC) after surgery using the hazard function and identify patients who might benefit from adjuvant chemotherapy.

**Patients and Methods:**

This retrospective study enrolled 994 patients with early-stage NSCLC who underwent radical surgical resection between January 1999 and October 2009. Survival curves were generated using the Kaplan-Meier method, and the annual recurrence hazard was estimated using the hazard function.

**Results:**

The median recurrence-free survival (RFS) was 8.8 years. The life table survival analysis showed that the 1-year, 3-year, 5-year and 10-year recurrence rates were 82.0%, 67.0%, 59.0% and 48.0%, respectively. Approximately 256 (25.7%) patients experienced relapse [locoregional: 32 (3.2%) and distant: 224 (22.5%)], and 162 patients died from cancer. The annual recurrence hazard curve for the entire population showed that the first major recurrence surge reached a maximum 1.6 years after surgery. The curve subsequently declined until reaching a nadir at 7.2 years. A second peak occurred at 8.8 years. An analysis of clinical-pathological factors demonstrated that this double-peaked pattern was present in several subgroups.

**Conclusions:**

The presence of a double-peaked pattern indicates that there is a predictable temporal distribution of the recurrence hazard of early-stage NSCLC. The annual recurrence hazard may be an effective method of selecting patients at high risk of recurrence, who may benefit from adjuvant therapy.

## Introduction

Screening with low-dose computed tomography (CT) has resulted in a growing number of early-stage non-small cell lung cancer (NSCLC) diagnoses [1]. Although curative surgical resection is the current treatment of choice for early-stage NSCLC, the risk of locoregional and distant relapse remains high, at 22%–40% [2]–[4]. Thus, understanding the clinical course of postoperative recurrent disease is essential for guiding effective treatment.

In most studies, the risk of recurrence is analyzed using survival curves rather than hazard functions [5], [6]. The hazard function, which depicts the rate of recurrence at any point in time among the remaining at-risk individuals, has been applied to provide insights into the patterns of recurrence of breast cancer [7], [8] and gastric cancer [9]. The hazard function describes not only the magnitude of the recurrence rate but also how it changes over time [7], [10].

To our knowledge, the present work is the first study of the temporal distribution of tumor recurrence hazard conducted with NSCLC patients. The temporal distribution of the recurrence hazard of early-stage NSCLC may shed light on the pattern of recurrence of NSCLC and may help identify patients who might benefit from adjuvant chemotherapy.

## Patients and Methods

### Institutional Review Board (IRB)

This study was approved by the Institutional Review Board of Sun Yat-sen University Cancer Center for the use of tumor samples and patients’ clinical history.

### Patients’ selection

A total of 994 primary NSCLC patients who underwent radical resection at the Sun Yat-sen University Cancer Center between January 1999 and October 2009 were eligible for inclusion in this study. All patients’ were staged or restaged as T1a-2bN0M0 according to the seventh edition of the International Union Against Cancer Staging System for Lung Cancer that was released in 2007 [11]. Non-smokers were defined as having smoked fewer than 100 cigarettes in their life time [12]. The cases selected for this study fulfilled the following criteria: (1) histological confirmed primary NSCLC; (2) no evidence of metastatic disease, as determined by history, physical examination and routine computed tomography (CT); (3) complete surgical resection (R0) at our cancer center; and (4) at least 3 months of follow-up information concerning disease recurrence and death. Patients who underwent non-curative resection (R1) or neo-adjuvant therapy and those who died of postoperative complications were excluded from the study.

There were 147 patients received platinum-based adjuvant chemotherapy within 4 to 8 weeks after surgical resection. Among these patients, 91 patients were in stage Ib and 56 patients in stage IIa. The average length of time between surgery and start of chemotherapy was 5.7 weeks. Among the patients who received adjuvant chemotherapy, four cycles of cisplatin (75 mg/m2) with paclitaxel or vinorelbine chemotherapy were performed in 94 (63.9%) patients and four cycles of carboplatin (an area under the curve dose of 6 mg/mL per minute over 60 minutes) with paclitaxel or vinorelbine were performed in the remaining 53 (36.1%) patients.

All participants had signed the written informed consent for their clinical records to be used in future study before starting initial treatment.

### Definition of recurrence

Local-regional recurrences were defined as those at the surgical site, at the anastomotic or bronchial stump, or in the local-regional lymph nodes (levels 1–14, including supraclavicular). Distant recurrences were defined as hematogenous metastases within solid organs, such as the lung, liver, brain and bone. Cervical or abdominal lymph node disease was considered a distant recurrence. Recurrences were diagnosed histologically, cytologically, and radiologically. A combined recurrence was defined as the detection of both locoregional and distant recurrences either simultaneously or within 30 days [13].

### Follow-up of patients

The patients were routinely followed up at the Cancer Center of Sun Yat-sen University every 3 months during the first year after surgery, every 6 months during the next 2 years and once per year thereafter. Follow-up was maintained through the retrieval of follow-up medical records stored in the outpatient department database or followed by personal contact with the patients by our professional follow-up institution, including requests for information regarding tumor recurrences and survival status. Recurrence or its absence was diagnosed by queries to the patient, chest CT, abdominal CT, bone scans, whole-brain CT/MRI or PET/CT. If a tumor had recurred, additional information, including sites of recurrence and therapy, was requested. All of the patients were contacted again in January 2013 to determine their vital status.

### Statistical analysis

Recurrence-free survival (RFS) was defined as the time from surgery to the earliest occurrence of relapse (locoregional or distant) or death from cancer or cancer-related disease. Patients who were lost to follow-up were censored at the time of last contact. Patients who were alive at the end of the study were censored for the purpose of data analysis. RFS was assessed using the Kaplan-Meier method and was compared using the log-rank test. The Cox regression model was used to perform a multivariate survival analysis of all of the variables that were significant in the univariate analysis. For the graphical display of RFS, the annual hazard rates were estimated using a kernel smoothing method. A two-sided probability value of less than 0.05 was considered statistically significant. All the statistical analyses were performed using the Stata statistical software package (release 9.0; Stata Corporation, College Station, TX, USA). The relative risks (RRs) are presented with their 95% confidence intervals (CIs).

## Results

### Patient characteristics

A total of 994 cases satisfied the inclusion criteria and were included in this study. The clinical-pathological characteristics of the 994 patients are listed in Table 1. All of these patients received rigorous follow-up, with a median follow-up time of 6.1 years.

**Table 1:** Kaplan-Meier postoperative survival analysis (log-rank test) according to clinical-pathological factors of patients with early-stage NSCLC.

Variable	N	Recurrence-free survival	95% CI	P
	994	8.784	8.389–9.179	
Sex				
Male	702	8.604	8.133–9.076	0.193
Female	292	9.204	8.488–9.921	
Age (years)				
≤65	680	9.179	8.713–9.646	0.010
>65	314	7.593	6.897–8.289	
Smoking history				0.037
Non-smoker^1^	429	9.267	8.685–9.848	
Smoker	565	8.400	7.866–8.935	
Initial symptoms				0.015
Examination	419	9.359	8.761–9.957	
Symptoms^2^	575	8.377	7.857–8.897	
Pathological type				0.663
Squamous cell carcinoma	319	8.859	8.259–9.214	
Non-squamous cell carcinoma	675	8.733	8.256–9.210	
Visceral pleural invasion				0.003
Present	392	8.249	7.598–8.901	
Absent	602	9.171	8.679–9.662	
Tumor diameter				<0.001
≤4.0 cm	759	9.138	8.691–9.585	
>4.0 cm	235	7.659	6.845–8.473	
Stations of resected lymph nodes				0.064
<6		8.575	8.111–9.038	
≥6		9.281	8.537–10.025	
Number of resected lymph nodes				0.022
<15	471	8.325	7.777–8.873	
≥15	523	9.265	8.700–9.830	
Lobar bronchus invasion				0.909
Present	181	8.922	7.844–10.000	
Absent	813	8.760	8.330–9.189	
Histological grade				0.012
G1+G2	631	9.111	8.619–9.602	
G3+G4	363	8.159	7.503–8.815	
CEA				<0.001
≤5 g/l	565	9.110	8.582–9.638	
>5 g/l	248	7.667	6.900–8.434	
Adjuvant chemotherapy^3^				0.321
Yes	147	8.681	8.255–9.107	
No	847	9.574	8.618–10.530	
Pathological T category				<0.001
P T1a	172	10.032	9.162–10.902	
P T1b	147	9.550	8.600–10.501	
P T2a	568	8.473	7.938–9.008	
P T2b	107	7.079	5.911–8.248	
AJCC stage				<0.001
P Ia stage	319	9.832	9.186–10.478	
P Ib stage	568	8.473	7.938–9.008	
P IIa stage	107	7.079	5.911–8.248	

1 Non- smokers were defined as having smoked fewer than 100 cigarettes in their lifetime; ^2^Common symptoms of lung cancer: cough, dyspnea, weight loss and chest pain; ^3^Postoperative cisplatin-based chemotherapy.

### Overall recurrence patterns

**Figure 1:**
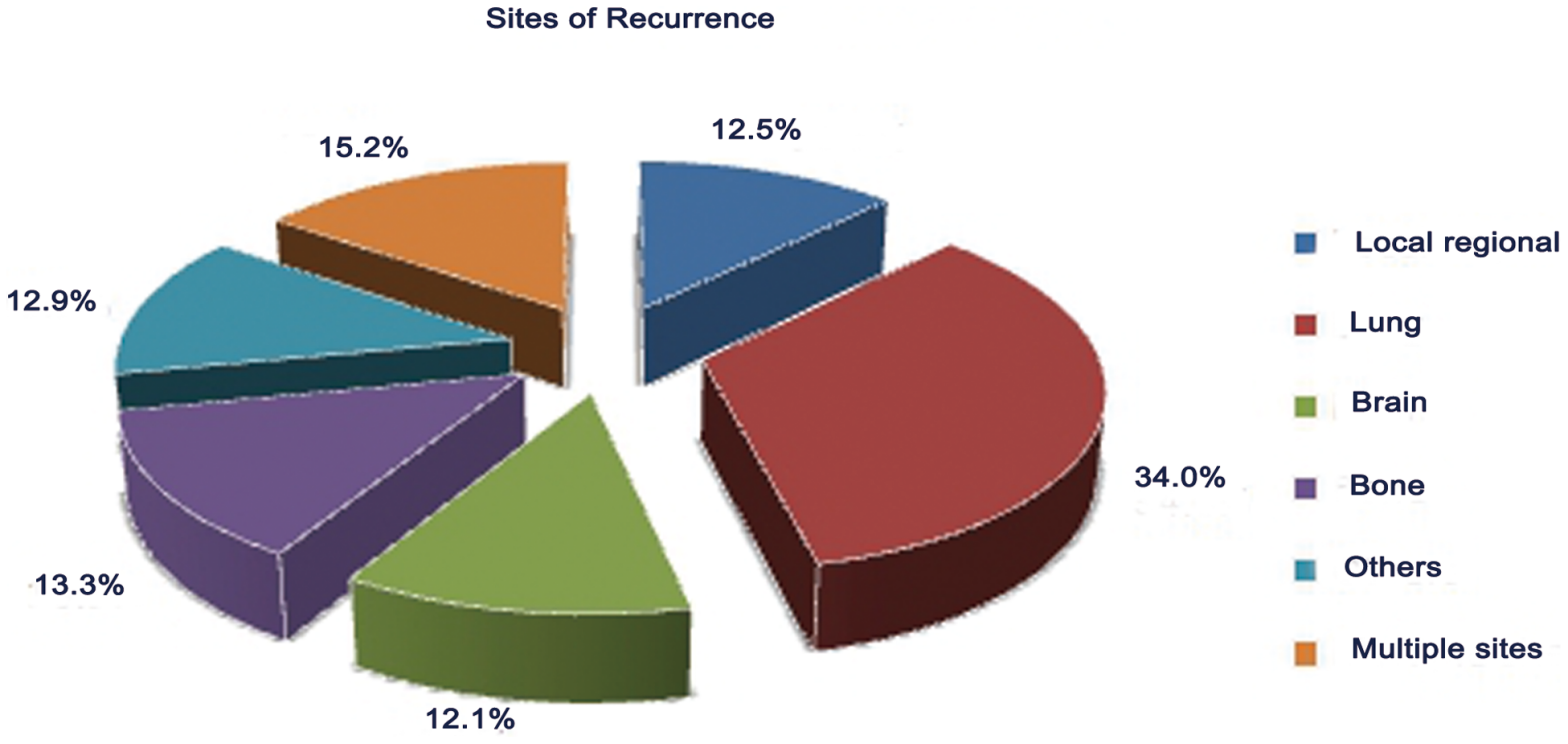
Relative frequencies for recurrence sites for postoperative early-stage NSCLC.

Approximately 256 patients experienced relapse (locoregional: 32 and distant: 224). The most common sites of failure were distant (87.5%, 224/256) in this group of patients. The most common site of distant metastasis was the lung (34.0%, 87/256), followed by the bone (13.3%, 34/256) (Figure 1). Overall, 162 patients died from cancer, and approximately 576 patients remained alive at the last follow-up.

### Survival analysis according to clinical-pathological factors

**Figure 2:**
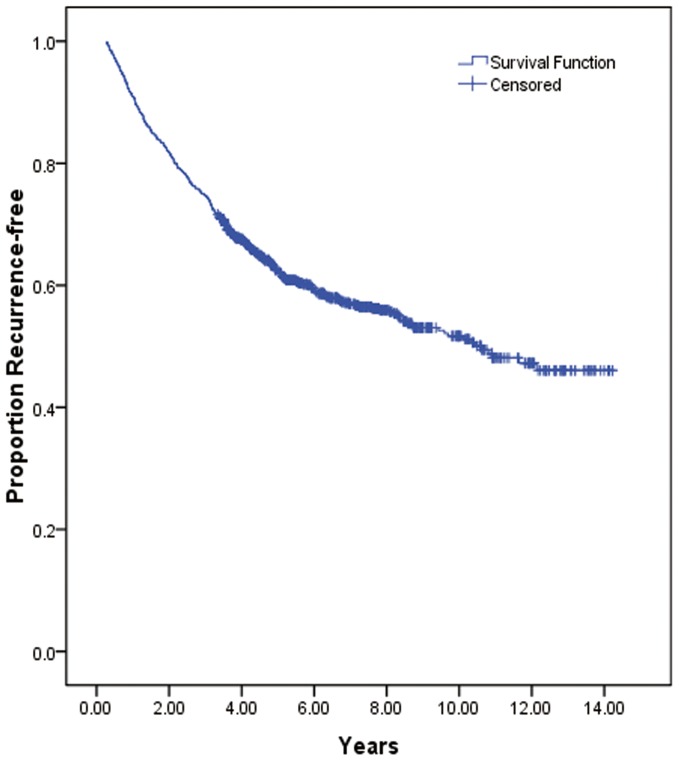
Recurrence-free survival for patients in early-stage NSCLC.

The survival curve for the entire population declined the most rapidly between 0.5 year and 2.5 years after surgery, with an extremely steep slope during this period. Subsequently, the entire curve continued a downward trend, albeit with a gentler slope, and it reached a virtual plateau by 10 years (Figure 2).

**Figure 3:**
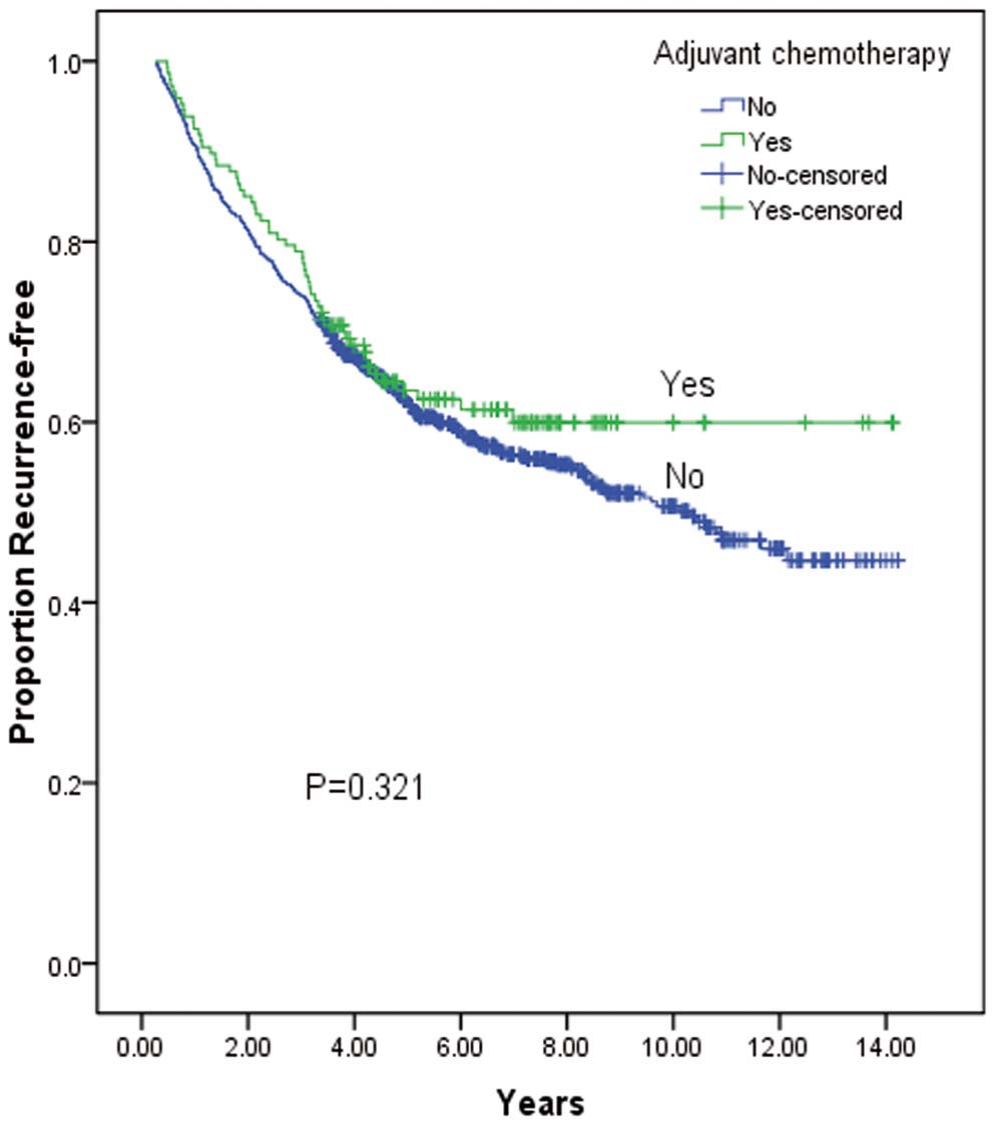
Recurrence-free survival for patients in early-stage NSCLC according to adjuvant chemotherapy.

The median RFS was 8.8 years. The life table survival analysis showed that the 1-year, 3-year, 5-year, and 10-year recurrence rates were 82.0%, 67.0%, 59.0% and 48.0%, respectively. The univariate analysis revealed that the following variables were significantly correlated with RFS: patient age, smoking history, initial symptoms, visceral pleural invasion, tumor diameter, number of resected lymph nodes, histological grade, level of carcinoembryonic antigen (CEA), and pathological T category (Table 1). The patients who received platinum-based adjuvant chemotherapy showed a longer RFS compared with those who did not (median 9.6 years versus 8.7 years); however, this difference was not statistically significant (Table 2) (Figure 3).

**Table 2:** Results of multivariate survival analyses of Recurrence-free survival (RFS) according to the Cox regression model.

Variable	N	RR	95% CI	P value
Tumor diameter		1.323	1.000–1.750	0.050
≤4.0 cm	759			
>4.0 cm	235			
CEA		1.414	1.131–1.768	0.002
≤5 g/l	565			
>5 g/l	248			
Number of dissected lymph nodes		0.781	0.630–0.968	0.024
<15	471			
≥15	523			
Age (years)		1.191	0.949–1.495	0.132
≤65	680			
>65	314			
Smoking history		1.003	0.795–1.264	0.983
Non-smoker	429			
Smoker	565			
Initial symptoms		1.137	0.904–1.429	0.273
Examination	419			
Symptoms	575			
Histological grade		0.824	0.655–1.036	0.097
G1+G2	631			
G3+G4	363			
Visceral pleural invasion		1.160	0.896–1.503	0.260
Present	392			
Absent	602			
Pathological T category		1.191	1.026–1.384	0.022
PT1a	172			
PT1b	147			
PT2a	568			
PT2b	107			

RR: relative risk; 95%CI: 95% confidence interval.

When the above variables were included in the multivariate analysis, the results suggested that tumor diameter, number of lymph nodes, CEA level, and pathological T category were independent factors that affected RFS (Table 2).

### Recurrence hazard analysis according to clinical-pathological factors

**Figure 4:**
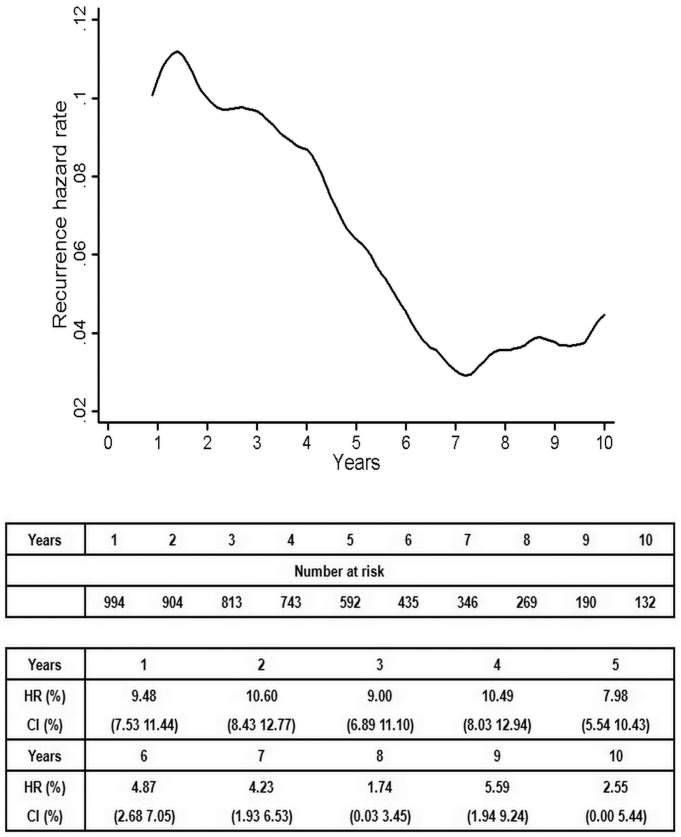
Annual recurrence hazard rates for patients in early-stage NSCLC.

The annual recurrence hazard curve for the entire population showed that the first major recurrence surge peaked 1.6 years after surgery. Subsequently, the curve declined until reaching a nadir at 7.2 years. A second peak occurred at 8.8 years (Figure 4). The time-varying patterns of recurrence after surgery are listed in Table 3.

**Table 3:** Patterns of recurrence after surgery.

Years after surgery	n	N		Type of recurrence		
			Locoregional	Distant	Death	
0–1	90	994	7	59	24	0
1–2	91	904	8	44	39	0
2–3	70	813	7	36	27	0
3–4	70	743	4	38	28	81
4–5	41	592	4	22	15	116
5–6	19	435	1	9	9	70
6–7	13	346	1	6	6	64
7–8	4	269	0	2	2	75
8–9	9	190	0	1	8	49
9–10	3	132	0	2	1	26
≥10	8	103	0	5	3	95
	418		32	224	162	576

**Figure 5:**
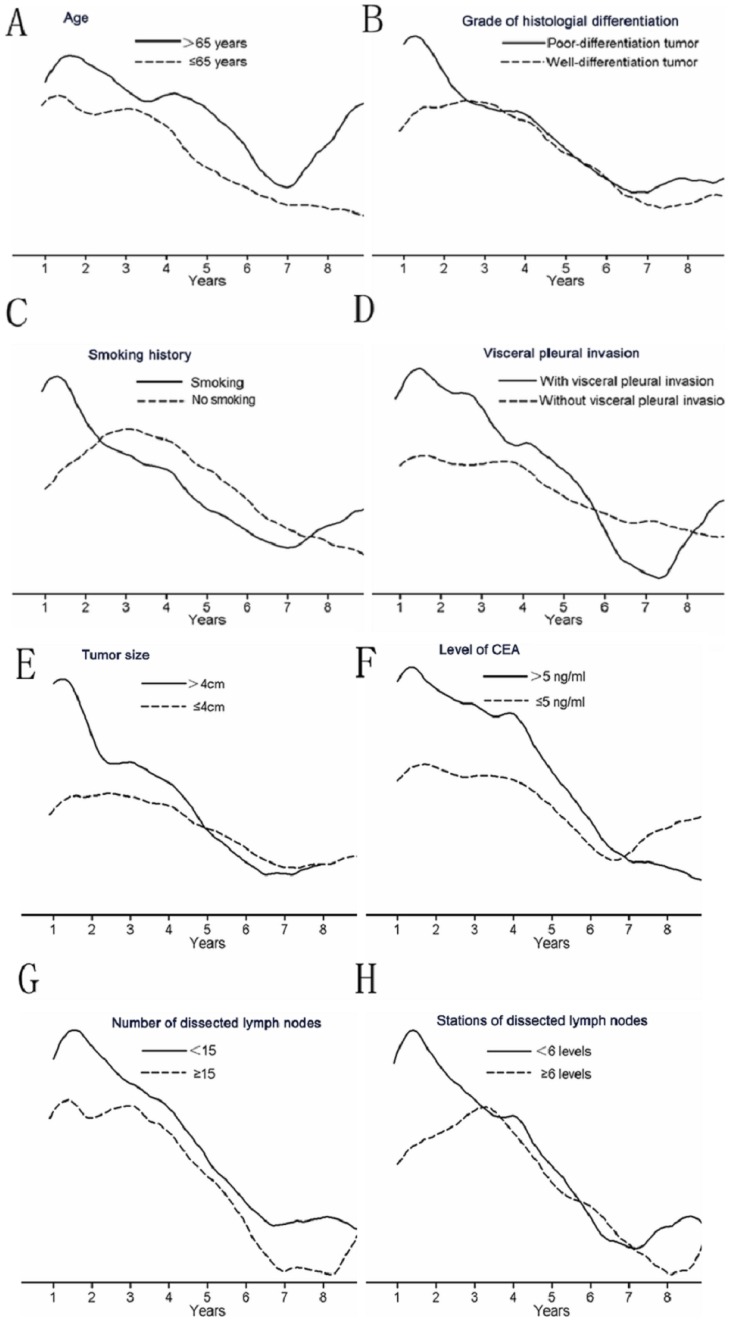
Recurrence hazard rates according to clinic-pathological characteristics. A: age; B: grade of histological differentiation; C: smoking history; D: visceral pleural invasion; E: tumor diameter; F: level of CEA; G: number of dissected lymph nodes; H: stations of dissected lymph nodes.

The temporal distribution of the recurrence risk varied with age. The older (≥65 years) patients exhibited a relatively pronounced and variable pattern compared with the younger (<65 years) ones. The smoothed hazard plots of these two subgroups were parallel to each other, with lower hazard rates in the younger (<65 years) patients (Figure 5A). The curve of the older (≥65 years) patients increased almost linearly after 7 years.

A visual inspection of the hazard curves for different grades of histological differentiation (Figure 5B) suggested that the patients with poorly differentiated tumors exhibited earlier and higher first peaks compared with the patients with well-differentiated tumors. However, the curves of the two groups were nearly identical after 2.8 years.

The hazard rate curves of the smokers and non-smokers were virtually superimposable (Figure 5C). The first peak of the smokers was lower than that of the non-smokers. The risk of recurrence was significantly higher for the smokers than for the non-smokers after 7.5 years. A similar phenomenon was observed among the patients with visceral pleural invasion(Figure 5D). The first peak of the patients without visceral pleural invasion appeared lower and later than that of the patients with visceral pleural invasion.

The analysis according to tumor diameter (≤4.0 cm versus >4.0 cm) demonstrated that the first recurrence peak was significantly lower and later for the patients with a tumor diameter of ≤4.0 cm compared with those with a tumor diameter of>4.0 cm(Figure 5E). The curves of both subgroups tended to intersect after 5.0 years, and there was no clear second peak in either group. The analysis according to CEA level (Figure 5F) revealed that although the patients with abnormal CEA level exhibited a higher first peak, the patients with normal CEA level tended to have a greater risk of recurrence after 6.8 years compared with the patients with abnormal level of CEA.

When the hazard rate was analyzed in terms of the number of dissected mediastinal lymph nodes, a double-peaked temporal distribution of recurrence was again revealed. The smoothed hazard plots of these two subgroups were parallel to each other, and the annual recurrence hazard curve (Figure 5G) demonstrated that the patients with <15 dissected lymph nodes tended to experience more relapses at all the analyzed time periods. In terms of the stations of the dissected mediastinal lymph nodes, the patients who were dissected at <6 stations exhibited both peaks at earlier time points(Figure 5H).

**Figure 6:**
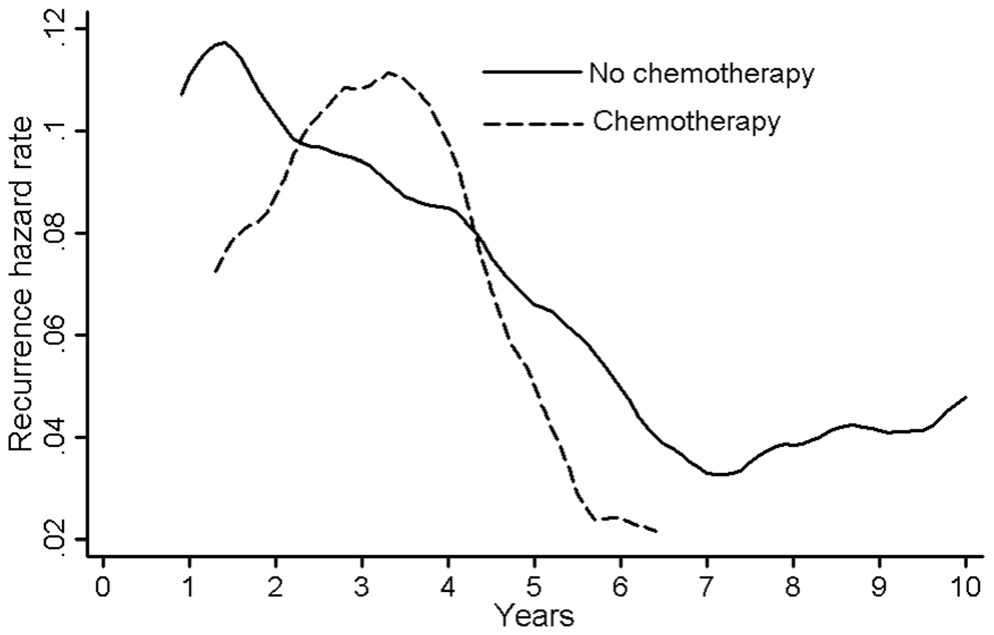
Recurrence hazard rates for patients in early-stage NSCLC according to adjuvant chemotherapy.

The type of treatment also affected the shape of the curves. Compared with those without adjuvant chemotherapy, the patients who received platinum-based adjuvant chemotherapy exhibited an later major recurrence surge. The first peak of the adjuvant chemotherapy-treated subgroup was 3.5 years after surgery, and the corresponding peak of their without chemotherapy-treated counterparts occurred at 1.5 years. After 4.2 years, the curve of the patients who received adjuvant chemotherapy was lower than that of the patients without adjuvant chemotherapy, and this pattern continued for the remainder of the study period (Figure 6).

## Discussion

Patients with early-stage (T1a-T2bN0M0) disease represent approximately 20%–30% of all patients with non-small cell lung cancer (NSCLC). Currently, surgery is the preferred treatment for early-stage NSCLC, and it is considered the only procedure with the potential to cure this condition [14]. However, the long-term survival of patients with early-stage NSCLC is still not optimistic. Despite surgical resection, approximately 20–40% of these patients die from local recurrence or distant metastasis within 5 years [2]. The present study is the first to demonstrate the presence of a double-peaked recurrence hazard pattern among early-stage (T1a-T2bN0M0) non-small cell lung cancer (NSCLC) patients after surgery. Our outcome of a double-peaked recurrence hazard pattern provides support for the theory of tumor dormancy [15], [16], which postulates that micrometastatic foci may exist in different biologic steady states, most of which do not promote tumor growth. However, this orderly and stable process may be perturbed by surgery, which stimulates a transition from dormancy to growth, resulting in a sudden acceleration of the metastatic process and eventually leading to recurrence [17]. This phenomenon may account for the first peak of recurrence risk of malignant carcinoma after surgery.

The site of recurrence of early-stage NSCLC after surgical resection was also investigated in this study. Among patients with early-stage NSCLC, most tumors recurred as distant metastases, rather than local-regional recurrences (22.5% versus 3.2%). In early-stage NSCLC after surgical resection, the rate of distant metastasis has been reported to be between 14.0% and 23.0% [18], [19], and the local-regional recurrence was 5.0% [20]. The patterns of tumor recurrence affect the therapy and survival of NSCLC patients. Based on the present study, the adjuvant treatment for early-stage NSCLC should be systemic therapy, rather than local therapy, due to the recurrence pattern of this disease.

High-risk factors, include poorly differentiated tumors [21], [22], vascular invasion [23], wedge resection [24], tumors >4.0 cm [25], visceral pleural involvement [26], [27], and incomplete lymph node sampling (Nx) [28], [29], are prognostic factors strongly associated with increased risks of recurrence and death among early-stage NSCLC patients. In our study, a double-peaked pattern was observed in a variety of patient subgroups, regardless of histological differentiation grade, tumor diameter, visceral pleural involvement or the number of dissected mediastinal lymph nodes; the patients with high-risk factors exhibited earlier and higher peaks. These factors should be considered when pondering treatment with adjuvant chemotherapy.

There is controversy concerning whether age affects the treatment and prognosis of lung cancer, particularly for patients with early lung cancer. Mery et al. [30] reported that age was an important prognostic factor for the survival of patients with stage I–II NSCLC after controlling for factors such as gender, histological type, clinical stage and type of surgery. Agarwal et al. [31] also confirmed that the mortality rate increased sharply with age in patients with stage I–II NSCLC: a one-year increase in age was associated with a nearly 6% increase in HR. However, some researchers have reported that age is not an important prognostic factor for the survival of early-stage lung cancer patients because elderly patients experience complications, organ impairment and other non-cancer-related factors [32], [33]. In the current study, older (≥65 years) patients exhibited a relatively pronounced and variable pattern compared with younger (<65 years) ones. The smoothed hazard plots of these two subgroups were parallel to each other, with lower hazard rates in the younger (<65 years) patients. The curve of the older (≥65 years) patients increased almost linearly after 7.0 years. Older age (≥65 years) may be an adverse prognostic indicator in early-stage NSCLC, especially among patients who live longer than 5 years after surgery.

In the present study, we verified that the double peaks were more prominent among patients who were smokers. The primary risk factor for NSCLC is tobacco smoking, which is involved in more than 85–90% of all lung cancer-related deaths [34], [35]. Although a history of smoking is well established as a risk factor for NSCLC, it remains controversial as a prognostic factor [36], [37]. In our data, the hazard rate curves of the non-smokers and smokers were virtually superimposable. The smokers showed an earlier major recurrence surge, with the first peak occurring 1.2 years after surgery, while the corresponding peak for their non-smoking counterparts occurred at 3.2 years. The risk of recurrence was significantly higher for the smokers than the non-smokers after 7.5 years.

Some studies have shown that level of CEA has prognostic significance in NSCLC [38], [39]. This finding was supported by our survival analysis, which showed that the risk of recurrence among patients with normal CEA level was significantly higher than that of the patients with abnormal CEA level before 7.0 years. However, the opposite pattern was observed after 7 years, indicating that the usefulness of CEA as a prognostic factor for recurrence risk may change over time. Thus, the CEA level tested before surgery may have significance for early recurrence, but not for late recurrence.

Many randomized clinical trials have reported the efficacy of platinum-based adjuvant chemotherapy after surgical resection in stage II-IIIA lung cancer [40]–[42]. However, the efficacy of platinum-based adjuvant chemotherapy in stage IB cancer is controversial [43], [44]. Pignon et al. performed a meta-analysis of the large adjuvant trials for NSCLC conducted since 1995 (excluding CALGB 9633) [45]. Their stage IB subset analysis (1,371 patients) trended toward showing a benefit of adjuvant treatment (HR, 0.93) but failed to reach statistical significance (95% CI, 0.78–1.10). In the current study, compared with those without adjuvant chemotherapy, the patients who received platinum-based adjuvant chemotherapy exhibited a first peak that appeared lower and later. After 4.2 years, the curve of the patients who received adjuvant chemotherapy was lower than that of the patients without adjuvant chemotherapy, and this pattern continued for the remainder of the study period. The results of our study demonstrate that for early-stage NSCLC patients, platinum-based adjuvant chemotherapy may reduce and delay the recurrence hazard.

The mechanisms for recurrence and metastasis of early stage NSCLC was unclear. Driver gene mutations were associated with the carcinogenesis and response to targeted therapies and prognosis of NSCLC. Some studies had reported that identification the driver gene mutation can select patients with early-stage disease who are at high risk of recurrence [46]–[47]. Brock et al. [48] also confirmed that methylation of the promoter region of the cyclin-dependent kinase inhibitor 2A gene p16, the H-cadherin gene CDH13, the Ras association domain family 1 gene RASSF1A, and the adenomatous polyposis coli gene APC in patients with stage I NSCLC treated with curative intent by means of surgery is associated with early recurrence.

Previous study reported that about 5.0% non small cell lung cancer have the risk of developing metachronous second primary lung cancer [49]. The standard diagnosis of second primary lung cancer was controversial, the different histological type from that of the first tumor was considered as the key criteria to define the second lung cancer. In our study, the initial data showed about 3.54% (39/1103) patients developed second malignant tumor during the flow-up after surgical resection: 10 patients developed second primary lung cancer, and 29 patients developed the other site malignant tumor. All these patients were diagnosed as secondary malignant tumor in histological type and were excluded from the present study. However, for patients with multiple tumors after surgical resection, our study was limited to distinguish the second malignant tumor from metastatic disease.

As a retrospective study, this study has other several potential limitations. The median follow-up time of 6.1 years was not sufficiently long; 576 patients were still alive at the last follow-up. Because there were only 147 patients who received platinum-based adjuvant chemotherapy, the subgroup analysis was unable to identify patients who might benefit from adjuvant chemotherapy.

In conclusion, we confirmed the validity of a double-peaked pattern of recurrence risk of early-stage (T1a-T2bN0M0) non-small cell lung cancer. The main pattern of tumor recurrence was distant metastasis, rather than local-regional recurrence. The hazard function may be useful for selecting patients at high risk of recurrence to receive postoperative therapy, which offers the possibility of decreasing and/or delaying the recurrence hazard.
